# Behavioural and medical predictors of bacterial vaginosis recurrence among female sex workers: longitudinal analysis from a randomized controlled trial

**DOI:** 10.1186/1471-2334-13-208

**Published:** 2013-05-08

**Authors:** Fernand A Guédou, Lut Van Damme, Jennifer Deese, Tania Crucitti, Marissa Becker, Florence Mirembe, Suniti Solomon, Michel Alary

**Affiliations:** 1 URESP, Centre de recherche FQRS du CHU de Québec, Département de Médecine Sociale et Préventive, Université Laval, Québec, Canada; 2 FHI360, Durham, NC, USA; 3 Department of Microbiology, Institute of Tropical Medicine, Antwerp, Belgium; 4 Centre for Global Public Health, University of Manitoba, Winnipeg, Canada; 5 Makerere University College of Health Sciences, Kampala, Uganda; 6 Y.R. Gaitonde Center for AIDS Research and Education, Chennai, India

**Keywords:** Bacterial vaginosis, Recurrence, Predictors, Female sex workers, Microbicide trial

## Abstract

**Background:**

Data on risk factors of recurrent bacterial vaginosis (RBV) are still scarce. We used data from female sex workers (FSW) participating in a randomized controlled microbicide trial to examine predictors of BV recurrence.

**Methods:**

Trial’s participants with at least an episode of BV which was treated and/or followed by a negative BV result and at least one subsequent visit offering BV testing were included in the analysis. Behavioural and medical data were collected monthly while laboratory testing for STI and genital tract infections were performed quarterly. The Andersen-Gill proportional hazards model was used to determine predictors of BV recurrence both in bivariate and multivariate analyses.

**Results:**

440 women were included and the incidence rate for RBV was 20.8 recurrences/100 person-months (95% confidence interval (CI) =18.1–23.4). In the multivariate analysis controlling for the study site, recent vaginal cleansing as reported at baseline with adjusted hazard-ratio (aHR)=1.30, 95% CI = 1.02-1.64 increased the risk of BV recurrence, whereas consistent condom use (CCU) with the primary partner (aHR=0.68, 95% CI=0.49-0.93) and vaginal candidiasis (aHR=0.70, 95% CI=0.53-0.93), both treated as time-dependent variables, were associated with decreased risk of RBV.

**Conclusion:**

This study confirms the importance of counselling high-risk women with RBV about the adverse effects of vaginal cleansing and the protective effects of condom use with all types of partners for the prevention of sexually transmitted infections, including BV. More prospective studies on risk factors of BV recurrence are warranted.

**Trial registration:**

Trial registration: NCT00153777

## Background

Bacterial vaginosis (BV) is the most common vaginal infection in women of reproductive age. Its prevalence varies from 9% to 50% [1,2] and may reach 70% among female sex workers (FSW) [3]. Besides its high frequency, BV is associated with many adverse health outcomes [4-10] including pelvic inflammatory disease, unfavourable pregnancy outcomes and recently HIV. BV is not only associated with female acquisition of HIV [9,10] but also with female-to-male transmission of HIV, as reported in a recent study [7]. Though it is increasingly clear that BV results from the replacement of the lactobacillus dominated normal flora by a predominantly anaerobic flora, no single causal agent has yet been identified. As a result, current treatment strategy aims at restoring the balance of the vaginal flora without specifically targeting any single causal agent. This may explain the high rate of treatment failure and recurrence that constitutes a major challenge for the clinical management and control of BV. Some authors [11-14] have suggested periodic presumptive treatment (PPT) as response to this challenge; however, the high recurrence rate makes the cost-effectiveness of this strategy questionable [15]. Knowing predictors of BV recurrence may help identify subgroups in whom PPT may be more efficient and prevent BV recurrences and their subsequent adverse health outcomes.

The purpose of this study was to identify behavioural and medical predictors of BV recurrence among women who experienced a prior BV episode during their follow-up at two African and two Indian sites of a randomized controlled microbicide trial.

## Methods

### The clinical trial

#### Settings and participants selection

We performed a secondary longitudinal analysis of data from HIV-negative FSWs enrolled in the cellulose sulphate (CS) trial, a double-blind randomized placebo-controlled trial evaluating the effect of 6% vaginal CS gel on HIV acquisition. Participant recruitment, follow-up and laboratory methods are described elsewhere [16]. Briefly, the trial recruited participants from five sites: Durban (South Africa); Kampala (Uganda); Cotonou (Benin); Chennai (India) and Bagalkot District (India). However, because of the occurrence of some local changes in the standardized method for BV laboratory diagnosis at the Durban site, and to ensure the comparability of our findings, the present analysis was restricted to participants from the latter four sites. Participants were FSWs who were 18 years or older, HIV seronegative, not pregnant and not desiring to become pregnant during their participation in the study. The study was approved by ethics committees (EC) of the Eastern Virginia School of Medicine, Norfolk, VA (USA), of the fhi360, NC (USA) and of each collaborating center: EC of the Centre hospitalier affilié universitaire de Québec in Canada, of the Faculté des Sciences de la Santé in Cotonou; of the National Aids Research Committee; of the Uganda Nation Research Council for Science and Technology; of YRG Care, Chennai, India; of the University of Manitoba, Winnipeg, Canada; of St. John's Medical College, Bangalore, India; of the Indian Council of Medical Research, Delhi, India and of the Medical Research Council, Durban, South Africa.

Trial participants provided written informed consent. At the screening visit, a questionnaire was administered to consenting women by trained health providers, asking about their socio-demographics, current contraceptive use, intra-vaginal cleansing practices (products used and reason), past history of STI and sexual behaviours (detail with the Additional file 1). Participants were then screened for HIV, syphilis, *Neisseria gonorrhoea*e, *Chlamydia trachomatis*, BV, *Trichomonas vaginalis* and vaginal yeast. All procedures, with the exception of the baseline questionnaire, were repeated at the enrolment visit (occurred within 28 days of screening). At enrolment, eligible women were randomized to active or placebo vaginal gel and were instructed to use their gel before each sexual act for 12 months. The participants received intensive counselling about condom use and were provided free condoms at each visit. Monthly behavioural data were collected (detail with Additional file 2) and pelvic exams with laboratory testing for HIV and other STI were performed quarterly. Follow-up was planned for 12 months, but the trial was closed prematurely following an interim analysis which suggested an increased risk of HIV among women in the active arm.

#### Laboratory procedures

BV diagnosis was made locally at each study site, by Gram stain, using the Nugent’s scoring system [17] and according to a standardised operation procedure (SOP). Trichomoniasis and candidiasis were diagnosed microscopically on wet mount. The endocervical swabs were tested with nucleic acid amplification tests for genital gonococcal and chlamydial infections. For syphilis diagnosis, blood samples were screened with Rapid Plasma Reagin (RPR) test and reactive samples were confirmed with a treponemal antibody test. At the screening and enrolment visits, HIV antibody testing was performed using the national HIV testing guidelines in place at each study site. For the follow-up visits, the study-specific algorithm was used. Pregnancy tests brands varied by site. All assays were conducted according to the manufacturers’ instructions. For all curable genital tract infections which were diagnosed, including BV, women with symptoms were treated at the same visit, whether lab test result was available or not (using the syndromic approach) while others were treated once test results became available, mostly in less than a week.

### Definitions of some concepts and study outcome for the present study

#### BV treatment

For the purpose of the present study, a BV treatment was deemed “effective” if it was in compliance with the 2006 UK guidelines for BV treatment [18].

#### Positive BV result

A positive BV result was defined as a Nugent score ≥ 7. Participants were defined as BV negative if they had a negative test result (Nugent score < 7) or were at least at the seventh day of the onset of an “effective BV treatment”.

#### Incident BV

Incident BV was defined as a positive BV result obtained at a follow-up visit (whether scheduled or not) which was at least 7 days from the onset of “effective BV treatment”, or following a negative BV result. A case of incident BV was assumed to have occurred at the midpoint between the date of the last negative BV result and the date of the visit at which the incident BV was diagnosed.

#### BV episode

A BV episode was defined as one or multiple consecutive positive BV results starting with the occurrence of an incident BV, as defined above, and ending either at the seventh day of an “effective BV treatment” or at the midpoint between the date of the last positive result of the BV episode and the date of the first negative BV result (whichever came first).

#### Recurrent BV (study outcome)

Recurrent BV (RBV) was defined as incident BV (as described above) following a positive BV result obtained at a previous visit (including the enrolment visit). A participant could have multiple recurrences during the follow-up period.

#### At-risk period

Participants with prior BV (index BV) were considered at risk of a RBV for the period spanning from the end of the index BV episode to the beginning of the next BV episode, or to the end of their follow-up, whichever occurred first. In cases of multiple BV recurrences, periods between BV episodes were considered as at-risk periods.

### Participant selection for the present analysis

The present analysis involved trial participants who, during follow-up (including the enrolment visit), had at least one BV episode, followed by:

an “effective BV treatment” and at least one subsequent visit at which a Nugent test was performed or;

a visit at which a negative BV result was obtained.

### Statistical methods

Participants’ baseline and follow-up characteristics are presented as percentages for categorical variables or medians along with inter-quartile ranges (IQR) for continuous variables. The RBV rate was obtained by dividing the number of RBV by the total number of person-months at risk. The 95% confidence intervals (95% CI) were obtained for the rate by bootstrapping 1000 samples with replacement. Variables reported in previous studies as potential predictors of RBV were selected for the analysis. These included socio-demographic characteristics, sexual behaviours, past history of, and current STI or reproductive tract infections.

Andersen-Gill proportional hazard models were used to identify independent predictors of RBV (as multiple events) by estimating hazard ratios (HR) [19]. The 95% CI of the HR and the p-values based on these models were derived from robust sandwich variances to account for intra-subject dependency attributable to the multiple recurrences that participants could experience [19].

Univariate models were fit with and without control for study site. Variables with p-value ≤ 0.20 in the univariate models controlling for study site were selected to construct the initial (full) multivariate model. Backward selection was used and variables which reached a significance level of 0.05 were retained in the final multivariate model as predictors of BV recurrence. Proportionality and linearity hypotheses were verified for the models. Statistical tests were two-tailed. Data were analysed with SAS software version 9.3 (SAS Institute, Cary, NC).

## Results

### Baseline socio-demographic, behavioural and biological characteristics of the participants

From July 2005 to January 2007, a total of 1491 women were screened and 822 were enrolled at the four study sites. Out of the 822, 440 women met the eligibility criteria for BV recurrence as described above and were included in the present analysis. Baseline socio-demographic, behavioural and biological characteristics of the participants are summarized in Table 1.

**Table 1:** Baseline characteristics of 440 female sex workers followed-up in a microbicide trial at 2 african and 2 indian sites

**Characteristics**	n with (%) or median with [IQR*]
Sites:	
Kampala	167 (37.9)
Cotonou	140 (31.8)
Indian sites (Chennai and Bagalkot District)	133 (30.2)
Age in years	28 [23-35]
Completed years of school	7 [3-9]
Cohabiting with a man	94 (21.4)
Exerting an occupation other than commercial sex work	128 (29.1)
Currently used contraceptive:	
None	255 (57.9)
Oral	29 (6.6)
Injectable	35 (7.9)
Intra-uterine device	3 (0.7)
Female sterilization	118 (26.8)
Having a primary partner	331 (75.2)
Number of sexual partners in the past 7 days†	8 [4 – 21]
Percentage of sexual act with primary partner using condom 100% ^‡^	97 (46.6)
Percentage of sexual act with other partners using condom 100% ^¶^	361 (97.6)
Oral or anal sex in the past 30 days with primary partner ©	15 (5.8)
Oral or anal sex in the past 30 days with primary partner without condom ©	10 (4.2)
Oral or anal sex in the past 30 days with other partners #	22 (5.0)
Oral or anal sex in the past 30 days with other partners without condom #	8 (1.4)
History of STI at baseline	321 (72.9)
Recent intra-vaginal cleansing (within 90 days) @	73 (17.6)
Irregular menstrual cycles	97 (22.0)
Laboratory diagnosis of STI or reproductive tract infections at baseline:	
Gonorrhoea ^§^	27 (6.2)
Chlamydia ^§^	28 (6.4)
Trichomoniasis ¥	20 (4.6)
Candidiasis ¥	112 (25.6)

*IQR =Inter-quartile range; †= missing data for 29 woman; ‡= The denominator excludes 207 women who reported not having a primary partner with whom they had sex in the last 7 days and 25 other who did not report on condom use; ¶ = The denominator excludes 44 women who reported not having sex with other partners in the last 7 days and 26 other who did not report on condom use; ©The denominator excludes 74 who reported not having a primary partner and 25 others who did not report on anal or oral sex; # =The denominator excludes 27 who did not report on anal or oral sex. §= missing data for 3 woman; ¥= missing data for 2 women; @= missing data for 25 women.

The median age of the study population was 28 years [inter-quartile range (IQR) = 23-35] and the median number of years in school was 7 (IQR=3-9). One hundred and twenty-eight (29.1%) of the women reported an occupation besides commercial sex work. The median number of sexual partners in the seven days preceding their study entry was 8 (IQR=4-21). Seventy-three (17.6%) reported recent (within 90 days) intra-vaginal-cleansing.

### Incidence of RBV

Among the 440 women, 253 (57.5%) experienced at least one BV recurrence during follow-up: 165 had one recurrence; 64 had two; 18 had three and 6 had four (giving a total of 371 recurrences). The 371 BV recurrences occurred over a total at-risk-period of 1783.12 person-months (146.56 person-years), giving an incidence rate of 20.8 recurrences/100 person-months (95% CI=18.1 – 23.4) or 2.53 recurrences/person-year (95% CI=2.26 – 2.77).

### Factors associated with RBV in univariate analysis

#### Baseline factors associated with RBV in univariate analysis

In univariate analysis without control for the study site, several baseline factors were significantly associated with RBV, as presented in Table 2. For positive associations these include being older than 28 years, cohabiting with a man, exerting an occupation besides sex work, recent intra-vaginal cleansing [since the visit preceding that of baseline (for women with BV at the enrolment visit in the trial, the preceding visit was the screening visit)], having an STI history and having had oral or anal sex with other partners in the 30 days prior to the baseline visit. In contrast, having been to school for at least six years, consistent condom use (CCU) with the primary partner in the past seven days and surprisingly, higher number of sexual partners, were inversely associated with RBV. There was a trend towards a positive association between use of intra-uterine device and RBV, and a negative association between hormonal contraception and RBV, but neither was statistically significant. Treatment group was not associated with BV recurrence.

**Table 2:** Association between baseline characteristics and incidence of bacterial vaginosis recurrence among 440 female sex workers followed-up in a microbicide trial: hazard ratios (unadjusted vs. adjusted for the study site)

**Baseline factors**	Incidence rate of BV recurrence (per 100 person-months) by exposure level (NR*/person-months)	**Unadjusted HR§ with 95% CI**^**¶**^	**p-value**	**HR§ (adjusted for study site) with 95% CI**^**¶**^	**p-value**
Study sites					
Chennai/ Bagalkot District	39.5 (142/359.57)	5.27 (3.90 – 7.12)	<0.0001	-	-
Cotonou	28.9 (160/553.93)	3.69 (2.76 – 4.94)	<0.0001	-	-
Kampala (ref)	7.9 (69/869.62)	1.00 -	-	-	-
Age (years; continuous)	-	1.03 (1.01 – 1.04)	0.0004	0.99 (0.97 - 1.00)	0.1452
Age (category):					
≥28 years (median)	25.8 (196/760.83)	1.52 (1.19 – 1.94)	0.0009	0.83 (0.64 - 1.07)	0.1476
<28 years	17.1 (175/1022.28)	1.00 -	-	1.00 -	-
Number of years in school (continuous)	-	0.99 (0.95 – 1.03)	0.6279	1.02 (0.98 - 1.05)	0.2832
Years in school (category):					
≥ 6 years	16.8 (151/900.52)	0.67 (0.52 – 0.86)	0.0015	1.05 (0.84 - 1.32)	0.6427
< 6 years	24.9 (220/882.60)	1.00 -	-	1.00 -	-
Living with a man					
Yes	31.9 (95/297.70)	1.75 (1.35 – 2.26)	<0.0001	0.99 (0.73 - 1.32)	0.9227
No	18.6 (276/1485.42)	1.00 -	-	1.00 -	-
Exerting an occupation besides sex work					
Yes	26.9 (123/457.67)	1.44 (1.10 - 1.89)	0.0076	0.89 (0.68 - 1.15)	
No (Ref.)	18.7 (248/1325.45)	1.00 -	-	1.00 -	0.3612
Having ever been pregnant					
Yes	21.3 (354/1659.72)	1.55 (0.89 – 2.71)	0.1201	1.16 (0.73 - 1.83)	0.5249
No	13.8 (17/123.40)	1.00 -	-	1.00 -	-
Current contraceptive method					
Female sterilization	38.2 (121/316.67)	2.21 (1.69 – 2.90)	<0.0001	0.99 (0.66 - 1.51)	0.9934
Intra-uterine device	37.9 (4/10.55)	2.16 (0.90 – 5.15)	0.0837	1.14 (0.56 - 2.34)	0.7215
Injectable	11.8 (18/152.70)	0.66 (0.39 - 1.13)	0.1308	1.21 (0.69 - 2.10)	0.5095
Oral	13.1 (18/137.25)	0.73 (0.41 - 1.29)	0.2800	0.98 (0.57 - 1.70)	0.9577
None (Ref.)	18.0 (210/1165.95)	1.00 -	-	1.00 -	-
Recent intra-vaginal cleansing reported at baseline					
Yes	32.3 (112/347.20)	1.75 (1.34 - 2.28)	<.0001	1.31 (1.03 - 1.66)	0.0279
No	18.5 (246/1326.27)	1.00 -	-	1.00 -	-
Number of sexual partners /last 7days (Continuous)	-	0.98 (0.97 - 0.99)	0.0003	0.99 (0.99 - 1.00)	0.4145
Number of sexual partners /last 7 days:					
≥ 8 partners (median)	14.9 (159/1067.18)	0.49 (0.39 – 0.63)	<0.0001	0.89 (0.67 - 1.18)	0.4079
< 8 partners	29.6 (212/715.93)	1.00 -	-	1.00 -	-
Having a primary partner					
Yes	21.6 (289/1337.92)	1.18 (0.88 – 1.59)	0.2679	0.98 (0.75 - 1.27)	0.8825
No	18.4 (82/445.20)	1.00 -	-		-
CCU‡ with primary partner in the past 7 days:					
Yes	17.3 (83/479.37)	0.68 (0.48 – 0.96)	0.0277	0.77 (0.56 – 1.07)	0.1236
No (ref.)	28.7 (134/466.28)	1.00 -	-	1.00 -	-
No primary partner or no sexual act with him	18.4 (154/837.47)	0.74 (0.55 – 0.99)	0.0459	0.97 (0.74 - 1.28)	0.8564
CCU‡ with other partners in the past 7 days:					
Yes	20.3 (329/1620.65)	0.49 (0.19 – 1.28)	0.1467	0.86 (0.34 – 2.18)	0.7505
No (ref.)	25.1 (19/75.70)	1.00 -	-	-	-
No sexual act with other partners	26.5 (23/86.77)	0.62 (0.22 – 1.71)	0.3556	0.96 (0.35 – 2.59)	0.9340
History of STI					
Yes	27.0 (176/651.55)	1.53 (1.14 - 2.05)	0.0046	1.13 (0.86 - 1.47)	0.3864
No	17.2 (195/1131.57)	1.00 -	-	1.00 -	-
Anal /oral sex with primary partner/ past 30 days					
Yes	29.2 (14/47.97)	1.30 (0.70 - 2.42)	0.4018	0.72 (0.38 - 1.34)	0.3011
No (Ref)	22.3 (292/1311.10)	1.00 -	-	1.00 -	-
Did not have primary partner	16.5 (52/314.40)	0.74 (0.52 - 1.04)	0.0800	0.99 (0.73 - 1.33)	0.9381
Anal /oral sex with primary partner without condom					
Yes	29.9 (9/30.08)	1.34 (0.68 - 2.65)	0.3951	0.78 (0.41 - 1.50)	0.4594
No (Ref)	22.3 (297/1328.98)	1.00 -	-	1.00 -	-
Did not have primary partner	16.5 (52/314.40)	0.73 (0.52 - 1.03)	0.0766	1.01 (0.74 - 1.35)	0.9931
Anal /oral sex with other partners/past 30 days					
Yes	46.4 (28/60.32)	2.26 (1.60 - 3.18)	<.0001	1.20 (0.82 - 1.74)	0.3434
No (Ref)	20.7 (330/1591.95)	1.00 -	-	1.00 -	-
Anal /oral sex with other partners without condom					
Yes	47.3 (10/21.15)	2.19 (1.08 - 4.44)	0.0298	1.17 (0.57 - 2.38)	0.6671
No anal or oral sex with other partners (Ref.)	21.3 (348/1631.12)	1.00 -	-	1.00 -	-
History of irregular menstrual cycles					
Yes	22.3 (78/349.37)	1.09 (0.80 – 1.48)	0.5733	1.11 (0.84 - 1.47)	0.4708
No	20.4 (293/1433.75)	1.00 -	-	1.00 -	-
Treatment group					
Active gel	21.5 (178/828.88)	1.07 (0.84 – 1.38)	0.5753	0.97 (0.77 - 1.21)	0.7645
Placebo	20.2 (193/954.23)	1.00 -	-	1.00 -	-
Gonorrhea					
Present	26.3 (31/117.82)	1.32 (0.81 - 2.12)	0.2618	1.27 (0.81 - 2.00)	0.30271
Absent	20.3 (336/1650.72)	1.00 -	-	1.00 -	-
Chlamydia					
Present	13.4 (18/134.65)	0.63 (0.34 - 1.15)	0.1319	1.07 (0.62 - 1.85)	0.8035
Absent	21.4 (349/1633.88)	1.00 -	-	1.00 -	-
Trichomoniasis					
Present	16.4 (14/85.27)	0.76 (0.35 - 1.62)	0.4720	0.90 (0.50 - 1.62)	0.7339
Absent	21.2 (357/1686.95)	1.00 -	-	1.00 -	-
Candidiasis					
Present	21.8 (85/389.47)	1.05 (0.80 - 1.39)	0.7130	0.97 (0.72 - 1.31)	0.8678
Absent	20.7 (286/1382.75)	1.00 -	-	1.00 -	-

*NR =number of recurrences; § HR=Hazard ratio; ^¶^ CI=Confidence interval; ‡ CCU=Consistent condom use (100% of sexual acts).

After controlling for study site, only recent intra-vaginal cleansing was significantly associated with RBV with an adjusted HR (aHR)=1.31, 95% CI=1.03 – 1.66.

#### Time-dependent factors associated with RBV in univariate analysis

Results from the univariate analysis of the associations between time-dependent variables and RBV are presented in Table 3. Oral sex, whether with the primary or other partners (whether all acts or those without condom use) was significantly and positively associated with RBV. In contrast, CCU with the primary partner, the presence of a vaginal candidiasis in the at-risk period and high number of sexual partners in the past seven days were inversely associated with RBV. For CCU with the primary partner and the presence of candidiasis, the association remained significant after controlling for study site.

**Table 3:** Association between time-varying factors and incidence of bacterial vaginosis recurrence among 440 female sex workers followed-up in a microbicide trial: hazard ratios (unadjusted vs. adjusted for the study site)

**Time-varying Factors**	Incidence rate of BV recurrence (per 100 person-months) by exposure level (NR*/person-months)	**HR§ and 95% CI**^**¶**^	**p-value**	**HR§ (adjusted for study site) with 95% CI**^**¶**^	**p-value**
Number of sexual partners/past 7 days (continuous)	-	0.99 (0.98-0.99)	0.0142	0.99 (0.99-1.00)	0.4145
CCU‡ with primary partner in the past 7 days:					
yes	18.8 (73/387.27)	0.58 (0.42-0.80)	0.0010	0.67 (0.49- 0.93)	0.0153
No (ref.)	32.3 (113/349.28)	1.00 -	-	1.00 -	-
No primary partner or no sexual act with him	17.6 (183/1039.67)	0.54 (0.42-0.71)	<0.0001	0.69 (0.53- 0.88)	0.0032
CCU‡ with other partners in the past 7 days:					
yes	18.8 (319/1457.60)	0.84 (0.41-1.72)	0.6353	1.33 (0.58-2.20)	0.7269
No (ref.)	24.9 (6/24.07)	1.00 -	-	1.00 -	-
No sexual act with other partners	16.0 (44/274.95)	0.62 (0.29-1.36)	0.2380	0.96 (0.47-1.97)	0.9077
Oral sex with primary partner/past 30 days					
yes	45.2 (11/24.35)	2.07 (1.24-3.47)	0.0054	1.10 (0.64 - 1.90)	0.7298
No	21.7 (324/1495.87)	1.00 -	-	1.00 -	-
No primary partner	13.8 (36/261.38)	0.62 (0.42-0.93)	0.0195	0.92 (0.65-1.28)	0.6094
Oral sex without condom with primary partner/past 30 days					
yes	46.8 (9/19.22)	2.24 (1.36-3.70)	0.0015	1.20 (0.71-2.03)	0.4934
No	21.7 (326/1501.00)	1.00 -	-	1.00 -	-
No primary partner	13.8 (36/261.38)	0.62 (0.42-0.92)	0.0190	0.92 (0.66-1.28)	0.6183
Oral sex with other partners/past 30 days					
yes	35.6 (17/47.72)	1.70 (1.16-2.50)	0.0062	0.86 (0.56-1.32)	0.4930
No	20.6 (353/1711.97)	1.00 -	-	1.00 -	-
Oral sex without condom with other partner/past 30 days					
yes	45.1 (4/8.87)	2.23 (0.90-5.52)	0.0824	1.19 (0.46-3.04)	0.7193
No	20.9 (366/1750.82)	1.00 -	-	1.00 -	-
Gonorrhea in the at-risk-period					
Present	24.5 (17/69.42)	1.18 (0.75-1.86)	0.4813	1.45 (0.87-2.416)	0.1482
Absent	20.7 (354/1713.70)	1.00 -	-	1.00 -	-
Chlamydia in the at-risk-period					
Present	16.1 (12/74.40)	0.77 (0.43-1.37)	0.3688	1.12 (0.67-1.87)	0.6720
Absent	21.0 (359/1708.72)	1.00 -	-	1.00 -	-
Trichomoniasis in the at-risk-period					
Present	15.7 (8/50.85)	0.73 (0.33-1.61)	0.4393	0.80 (0.37-1.71)	0.5589
Absent	20.9 (363/1732.27)	1.00 -	-	1.00 -	-
Candidiasis in the at-risk-period					
Present	15.6 (86/552.65)	0.68 (0.53-0.87)	0.0025	0.69 (0.53-0.91)	0.0095
Absent	23.2 (285/1230.47)	1.00 -	-	1.00 -	-

*NR =number of recurrences; § HR=Hazard ratio; ^¶^ CI=Confidence interval; ‡ CCU=Consistent condom use (100% of sexual acts).

### Factors associated with RBV in the multivariate analysis

Variables with p-value<0.20 in the univariate analysis controlling for study site were included in the multivariate model. Recent intra-vaginal cleansing as self-reported at the baseline visit was positively associated with RBV, whereas CCU with the primary partner in the past seven days and vaginal candidiasis in the at-risk period were protective (Table 4). In addition, study site remained significantly associated with RBV. None of the STI or genital tract infections, other than candidiasis, was significantly associated with RBV.

**Table 4:** Baseline and time-varying factors predicting bacterial vaginosis recurrence among 440 female sex workers followed-up in a microbicide trial: adjusted hazard ratios (Multivariate model)

**Factors**	**HR§ and 95% CI**^**¶**^	**p-value**
Study sites:		
Chennai/Bagalkot District	4.83 (3.49 – 6.68)	<0.0001
Cotonou	3.16 (2.30 - 4.33)	<0.0001
Kampala (Ref.)	1.00 -	-
CCU‡ with primary partner in the past 7 days:		
No	1.00 -	-
Yes	0.68 (0.49 - 0.93)	0.0169
No primary partner or no sexual act with him	0.72 (0.56 - 0.92)	0.0085
Recent intra-vaginal cleansing (as reported at baseline)	1.30 (1.02 - 1.64)	0.0322
Candidiasis in the at-risk-period	0.70 (0.53 - 0.93)	0.0130

§ HR=Hazard ratio; ^¶^ CI=Confidence interval; ‡ CCU=Consistent condom use (100% of sexual acts).

## Discussion

Among these 440 HIV seronegative FSW with a BV episode at baseline, we found that 253 (57.5%) have had at least one BV recurrence during their follow-up. The incidence rate was 20.8 recurrences/100 person-months (95% CI=18.1 – 23.4) or 2.53 recurrences/person-year (95% CI=2.26 – 2.77).

Most prior studies on RBV looked only at the first recurrence, in the context of treatment regimen evaluation [20-23]. Therefore, their primary outcome was the proportion of treated subjects experiencing at least one BV recurrence in a given post-treatment length of time. This makes it difficult to compare the RBV frequency in the present study to those previously reported.

The two studies which reported BV incidence as multiple events were sub-studies of the same trial that evaluated the effect of PPT on the incidence of vaginal infections [12]. The first study, which was a secondary analysis focusing on the placebo group only, found a BV incidence rate of 361/100 person-years over the trial period [24]. The second study evaluated the post-trial effect of the PPT (first 120 days post-trial) and found RBV incidence of 260/100 person-years (95% CI=199-340) and 358/100 person-years (95% CI=286-448) for the intervention and placebo groups, respectively [25]. The RBV incidence of 253/100 person-years in the present study is somewhat lower than the three others, though only significantly for the two results reported from the placebo group (whether in- or post-trial). This discrepancy may be due to the fact that, unlike in the current study where all microscopically diagnosed BV were treated, (regardless of the presence of symptoms), only symptomatic BV were treated in the PPT and post-PPT studies. These two studies may have thus reported several times some persistently asymptomatic and thus untreated BV. Consequently we may rather be comparing incidence of “visits with BV diagnosis” to that of RBV.

In the multivariate analysis controlling for study site, recent vaginal cleansing, as reported at study entry, was a risk factor for RBV, whereas CCU with the primary partner and vaginal candidiasis were negatively associated with it.

While several previous cross-sectional or prospective studies found that intra-vaginal cleansing increased the risk of single BV, very few dealt with recurrent BV [24,26,27]. Schwebke et al. reported that vaginal douching increased the risk of RBV [26], and the first sub-study on the PPT trial data found that the risk of RBV increased with vaginal washing frequency (p-value for trend=0.04) [24]. These findings are consistent with our results, though we did not collect data on vaginal washing frequency. However, some studies found no association between vaginal douching and BV [21,27]. In fact, although vaginal washing is a common practice, the frequency, techniques, products used and circumstances vary from one individual to another, and from culture to culture. This might explain the inconsistencies in studies results.

Compared to women who reported inconsistent condom use with their primary partners in the previous seven days, those who reported CCU were significantly at lower RBV risk, and interestingly their aHR was similar to that of women with no primary partner or no sexual act with the latter. These results support the idea that consistent and proper use of condoms may be as effective as sexual abstinence in protecting against BV recurrence. The non-significance of the association between CCU with other sexual partners (clients) and RBV may be due to a lack of statistical power resulting from the high percentage of condom use (97.6%) reported for this subgroup of sexual partners. Several studies have shown that CCU can reduce BV recurrence [24,26,28,29]. However, Yotebieng et al. found that condom use was protective against BV occurrence but not BV recurrence [30]. Unlike with a single BV episode, we did not find any association between STI or reproductive tract infections (except candidiasis) and RBV, and neither did Klatt et al. and Myer et al. [27,31]. However, in some studies, trichomoniasis was associated with RBV [24,32].

Vaginal candidiasis (VC) during follow-up (but not at baseline) was inversely associated with RBV, consistent with the results of McClelland et al. [24]. The common explanation is that the two conditions are incompatible as they are inversely related to vaginal pH. Another study found that VC often preceded a BV episode though they rarely coexisted [33]. The reason of such a chronology remains unknown.

Finally, the study site exhibited a consistently strong association with BV recurrence, independently of all other factors, suggesting that RBV may be determined by a more complex network of site-specific unmeasured and probably interrelated factors. A striking finding in the current study is the overwhelming effect of the study site on the associations between investigated risk factors and RBV. With the exception of recent vaginal cleansing as self-reported at baseline, CCU with the primary partner and vaginal candidiasis (as time-dependent variables), all the associations which were initially significant lost their significance when controlled for study site. This suggests strong confounding by site due to a highly variable distribution of socio-demographic and medical variables across sites. Nevertheless, the univariate association between anal or oral sex (receiving penis in the mouth) and RBV deserves some attention.

The role of oral or anal sex in BV occurrence is supported by several studies [32,34-37]. In a recent prospective study [35], women with incident BV were more likely to have previous colonization of anus or oral cavity with BV-associated bacteria. In another study [34], the risk of periodontal disease was increased among women with BV (adjusted risk ratio =1.23; 95% CI: 1.08-1.40). In the same study, the risk for periodontal disease was 1.28 times (95% CI: 0.97-1.69) greater for receptive oral sex (ROS) with an uncircumcised partner, compared with ROS with a circumcised partner [34]. In another study, having vaginal sex after receptive anal intercourse was linked with acquisition of BV [37].

The primary limitation of the current study resides in assuming for some BV episodes (in the absence of test of cure) that BV treatment, as administered per local guidelines, became effective seven days from the onset of the treatment. Some persistent BV may have thus been taken as RBV and this would have resulted in an overestimation of the RBV incidence. However the setting of the cure timeline of 7 days was based on the cure (Nugent score <7) rate of 80% to 90% generally reported in the literature for the same duration regarding the treatment regimens selected for our analysis [38]. Because of the relatively long periodicity of BV testing (3 months) we may have missed some BV episodes. We were not able to assess the effect of vaginal cleansing as reported during follow-up, because the proportion of women reporting the practice drastically declined over follow-up. This decrease resulted from the consistent counselling provided to women against vaginal cleansing at monthly visits as part of the instructions for the use of the study product. However, since data on vaginal cleansing were self-reported, those collected during follow-up, subsequently to several counselling sessions might be more subject to the effect of social desirability than those collected at study entry. Therefore, the former may be less appropriate than the latter in assessing the effect of vaginal cleansing on RBV. Also, some established BV risk factors, such as HSV-2 infection, smoking and alcohol consumption could not be included in the analysis because no data were collected about them in the trial. Finally, the current analysis did not cover biological predictors of RBV such as the presence and/or concentration of some specific BV related micro-organisms.

Nonetheless, this study presents several strengths. The primary one is the use of Andersen-Gill proportional risk modeling which allows for repeated events. To our best knowledge, the present study is the third analysis (after the first two by McClelland et al.) and the largest one that has used this approach to investigate predictors of RBV. Also, the consideration of BV clinical features to work out the at-risk period, instead of using the whole follow-up period, prevents underestimation of the RBV incidence. Like with other infectious diseases, the subject is not at risk for another BV episode until the previous resolves and this should be considered when calculating the person-time at risk (which was not done in previous studies). Other strengths include the large sample size, the use of the current gold standard for BV diagnosis (Nugent score) and the high level of the quality assurance system conferred by the clinical trial setting.

## Conclusions

In summary, from this longitudinal analysis of data from a randomized clinical trial, we report a relatively high rate of RBV, though lower than in some previous studies [24,25], among FSWs HIV seronegative at baseline. Some common risk factors for a single BV episode were associated with RBV while others were not. Predictors of RBV were primarily behavioural, particularly unprotected sex with primary sex partner and recent intravaginal cleansing. It is thus important to counsel high-risk women with RBV about the adverse effects of vaginal cleansing and the protective effects of condom use with all types of partners.

The study site exhibited a consistently strong association with RBV suggesting that research on risk factors of this condition may need to be as well approached as a socio-cultural environmental issue. Finally more prospective studies, specifically designed to identify risk factors of RBV are still warranted.

## Competing interest

None of the authors has any conflict of interest to declare.

## Authors’ contributions

All authors were involved in the parent multicenter microbicide clinical trial that generated the data. For the present manuscript, Fernand A. Guédou (FAG) and Michel Alary (MA) conducted the statistical analyses and interpreted results. FAG wrote the first draft of the manuscript and MA revised it before further revision by other co-authors. In addition, Jennifer Deese (JD) and Marissa Becker (MB) edited the text. All authors revised and approved the present version of the manuscript.

## Pre-publication history

The pre-publication history for this paper can be accessed here:

http://www.biomedcentral.com/1471-2334/13/208/prepub

## Supplementary Material

Additional file 1 Screening form, questionnaire administered at the screening visit. Click here for file

Additional file 2 Follow-up form, questionnaire administered at the follow-up visits. Click here for file
