# Supplementary material for: Behavioural and medical predictors of bacterial vaginosis recurrence among female sex workers: longitudinal analysis from a randomized controlled trial
Source: BMC Infect Dis. 2013 May 8;13:208. doi: 10.1186/1471-2334-13-208 (PMC3655069; doi:10.1186/1471-2334-13-208)
Supplement: Additional file 1 — Screening form, questionnaire administered at the screening visit. [file 1471-2334-13-208-S1.pdf]

FSN FORMTYPESCREEN

CONRAD CS Phase 3 Study

Screening Form

1. Study Number: |__9__|__8__|__4__|__5__| **STUDY**
2. Center Number: |____|____|____|____|____| **CN** CSHP_CENTER
3. Screening Number: |____|____|____|____| **PN**
4. Date of Visit: |____|____|/|____|____|/|____|____|____|____| CONDAY CONMTH CONYEAR **CONDATE**
   day month year
5. ID # of person completing form: |____|____| **SCID**
6. Participant signed the screening consent form ...|____|
   0=No → **STOP, participant must be consented**
   1=Yes **SCCONSEN** CSHP_NOYES
7. How old are you in years?... **SCAGE** …|____|____|

   If less than 18, participant is not eligible for study. Complete FINAL form after completing this form.
8. What is your marital status?. **SCMAR** …………|____|
   1=Not currently married, not living with man
   2=Not currently married, living with man
   3=Married, not living with man
   4=Married, living with man CSHP_MARITAL
9. How many years of school have you completed?. **SCEDU** ……………………...|____|____|
10. What is your occupation?. **SCOCC** ……………|____|
    0=None 3=Professional CSHP_OCCUP
    1=Student 4=Domestic
    2=Trade/commerce 5=Other → **specify**:
    __**SCOCCO** __ *Q10CODE1-Q10WORK* ______
11. Have you ever been pregnant? **SCPRG** ………|____|
    0=No → **Skip to item 14**
    1=Yes CSHP_NOYES
12. Date last pregnancy ended

    |____|____|/|____|____|/|____|____|____|____|
    Day Month Year
    SCPRDAY SCPRMTH SCPRYEAR **SCPRDATE**
13. Parity status
    a. Pregnancies (total number) **SCNPREG** |____|___|
    b. Vaginal deliveries (including stillbirths) |___|___|
    SCNVGDEL
14. Have you ever used a spermicide? ....|____|
    0=No **SCSPERM**
    1=Yes CSHP_NOYES
15. Current contraceptive use CSHP_NOYES (A-F)

(Answer 0=NO or 1=YES for each)

a. Oral. **SCORAL** …………………………|____|
b. Injectables. **SCINJ** ……………………..|____|
c. IUD. **SCIUD** ……………………………..|____|
d. Condom. **SCCON** ………………………|____|
e. Female sterilization. **SCFS** ……………|____|
f. Other → **Specify**: **SCOTHO _SCOTH** |____|
   Q15CODE1-Q15WORK

16. Do you douche (wash inside your vagina)? ....|____|
    0=No CSHP_NOYES **SCDOUCHE**
    1=Yes → **Specify**:
    a. What do you use? **SCDOUCHO** ____
       Q16ACOD1-Q16AWORK
    b. Why do you douche? **SCYDOUCO** ____
       Q16BCOD1-Q16BWORK
17. Have you ever had an STI?. **SCSTI** …………...|____|
    0=No CSHP_NOYES
    1=Yes → Specify:
    SCSTIO *Q17CODE1-Q17WORK* __

    How many different men have you had vaginal sex with in the last 3 months? |__|__|__|__|
    SC3MEN(Item SC3MEN was changed to a length of 4 in Clintrial production.)
18.

    If fewer than 3 times, participant is not eligible. Complete FINAL form after completing this form.
19. How many of these men whom you had vaginal sex with in the last 3 months were new sexual partners? **SC3NEW** ………………..|__|__|__|__|

    (Item SC3NEW was changed to a length of 4 in Clintrial production.)
20. On average, how many times do you have vaginal sex in a week (7 days)? |__|__|__|
    SCAVGSEX(Item SCAVGSEX was changed to a length of 3 in Clintrial production.)

    If less than 3 times, participant is not eligible. Complete FINAL form after completing this form.
21. How many times did you have vaginal sex in the last 7 days? **SC7SEX** ……………..|__|__|__|

    (Item SC7SEX was changed to a length of 3 in Clintrial production.)
22. The last time you had vaginal sex, did you use a condom?. **SCCLT** ……………………………….|____|

    0=No
    1=Yes CSHP_NOYES

Initials of person completing form: ____________ ***Date (dd/mm/yyyy)***: __________ _____

23. Have you had anal sex (penis in anus) or oral sex (penis in mouth) in the past 30 days?..................|____|

0=No SCANOR
1=Yes (anal)
2=Yes (oral) CSHP_NYSEX
3=Yes (both anal and oral)
