# Supplementary material for: Behavioural and medical predictors of bacterial vaginosis recurrence among female sex workers: longitudinal analysis from a randomized controlled trial
Source: BMC Infect Dis. 2013 May 8;13:208. doi: 10.1186/1471-2334-13-208 (PMC3655069; doi:10.1186/1471-2334-13-208)
Supplement: Additional file 2 — Follow-up form, questionnaire administered at the follow-up visits. [file 1471-2334-13-208-S2.pdf]

* Refer to Protocol or Study Manual for definitions of Relatedness

FORMTYPEFU

CONRAD CS Phase 3 Study

Follow-up Visit

1. Study Number: |__9__|__8__|__4__|__5__| **STUDY**
2. Center Number: |____|____|____|____|____| **CN** CSHP_CENTER
3. Screening Number: |____|____|____|____| **PN**
4. Date of Visit: |____|____|/|____|____|/|____|____|____|____| CONDAY CONMTH CONYEAR **CONDATE**
   day month year
5. ID # of person completing form: |____|____| **FUID**
6. Follow-up visit (01-12) **FUVNUM** …………|____|____|
7. Has participant experienced a new medical problem or worsening of an existing problem since last visit?... **FUMEDPRB** …………………………|____|
   0=No CSHP_NOYES
   1=Yes → **Specify FSN(s) of completed AE Form(s): FUAEFSNO** ____________________
8. Has participant experienced any genital bleeding other than menses since the last visit? ....|____|
   0=No CSHP_NOYES **FUGB**
   1=Yes → **Complete AE and IMB Forms.**

   FSN(s) of AE Form(s): FUGBFSNO __________

   FSN(s) of IMB Form(s): FUIMFSNO __________
9. Has participant used any intravaginal products other than study product since last visit? **FUINVAG** ….|____|
   0=No CSHP_NOYES CSHP_NOYES (A-E)
   1=Yes→ **Read each answer choice to participant and answer each item with 0=No or 1=Yes**
   a. Other spermicide. **FUOSPRM** …………|____|
   b. Non-study condom. **FUNSCON** ……….|____|
   c. Tampons. **FUTAMP** ……………………..|____|
   d. Douche. **FUDOUCHE** ……………………|____|
   e. Other → Specify: **FUOTHO** __..**FUOTH** |____|
   Q9CODE1-Q9WORK
10. Has participant taken any new medications since her last visit?..... **FUMED** …………………………|____|
    0=No CSHP_NOYES
    1=Yes → **Specify FSN(s) of completed CON Form(s): FUCTFSNO** ____________________
11. Has participant had problems using the gel **since her last visit**?.. **FUPGL** ……………………………….|____|
    0=No CSHP_NOYES
    1=Yes → **Specify: FUPGLO** ________________

    Q11CODE1-Q11WORK ________________

    If the problems were adverse events, please ensure item 7 is correct.
12. How many different men has the participant **FU7MEN** had vaginal sex with **in the last 7 days**? |__|__|__|

    (Item FU7MEN was changed to 3 in length in production.)
13. Participant status **FUPDISP** CSHP_PTSTAT….|____|
    1=Continue to next scheduled follow-up visit
    2=Discontinued at this visit → **Complete FINAL form**

Complete the following with regard to VAGINAL sex acts. Enter '00' where applicable.

**In the past 7 days: How many**	**With Primary Partner**	**With Other Partners**
14. Vaginal sex acts	**FU7SEXP** |__|__|__|	**FU7SEXO**|__|__|__|
15. Vaginal sex acts using a new study gel, but no new condom	**FU7GLP**|__|__|__|	**FU7GLO**|__|__|__|
16. Vaginal sex acts using a new condom, but no new study gel	**FU7CONP**|__|__|__|	**FU7CONO**|__|__|__|
17. Vaginal sex acts using both a new study gel and new condom	**FU7GLCNP**|__|__|__|	**FU7GLCNO**|__|__|__|

(Items FU7SEXP, FU7SEXO, FU7GLP, FU7GLO, FU7CONP, FU7CONO, FU7GLCNP, FU7GLCNO were changed to 3 in length in production.)

Complete the following with regard to ANAL and ORAL sex. Enter 0=No or 1=Yes in each box.

**In the past 30 days: Did you ever have**	**With Primary Partner**	**With Other Partners**
18. Anal sex	**FU30ASXP** |____|	**FU30ASXO** |____|
19. Anal sex without using a new condom	**FU30ACNP** |____|	**FU30ACNO** |____|
20. Oral sex	**FU30OSXP** |____|	**FU30OSXO** |____|
21. Oral sex without using a new condom	**FU30OCNP** |____|	**FU30OCNO** |____|
	CSHP_NOYES (18-21)	CSHP_NOYES (18-21)

***Initials of person completing form*: ________________ *Date (dd/mm/yyyy)*: ______________**
